# Modeling and Simulation of the Gasification of *Euterpe oleracea* Waste: Optimization of Hydrogen Production and Energy Potential through Operability Analysis

**DOI:** 10.1021/acsomega.6c01087

**Published:** 2026-06-01

**Authors:** Cassiano M. Musial, Júnior Staudt, Augusto Verdi Reichert, Edson A. da Silva, Caroline Ribeiro, Fernando V. Lima, Carlos E. Borba

**Affiliations:** † Postgraduate Program in Chemical Engineering, 201362 West Parana State University, Campus Toledo, Faculdade St. 645, Jd. La Salle, Toledo, Paraná 85903-000, Brazil; ‡ Laboratory of Chemical Process Engineering, Technical University of Munich, Campus Straubing for Biotechnology and Sustainability, Straubing 94315, Germany; § Department of Chemical and Biomedical Engineering, 5631 West Virginia University, Engineering Sciences Building, 1306 Evansdale Dr, Morgantown, West Virginia 26506, United States

## Abstract

Hydrogen production has been transitioning toward sustainable methods due to the increasing demand for carbon emission reduction. Biomass gasification has emerged as a promising alternative, converting organic waste into syngas that is rich in hydrogen and carbon monoxide under high-temperature conditions. This study focuses on the thermodynamic modeling and simulation of the gasification process using açaí seed residue as feedstock. The process model was implemented in Aspen Plus, considering key operating variables such as temperature, steam-to-biomass (S/B) ratio, and equivalence ratio (ER). Additionally, an operability analysis was conducted to optimize the gas composition, energy demand, and cold gas efficiency (CGE). Results indicated that a higher S/B ratio enhances hydrogen production due to the water–gas shift reaction, whereas an increased air supply improves energy efficiency but reduces hydrogen content in the output. The operability-based optimization revealed that the hydrogen concentration in syngas can reach up to 58.6% when no energy demand restrictions are applied, but at a high energy cost (444 MJ h^–1^). Conversely, the operability index increased from 13% to 30.2% by applying an energy self-sufficiency constraint (heat duty < 0), making the process more sustainable while maintaining CGE between 78.6% and 98.1%. These findings highlight the trade-off between syngas quality and energy efficiency, which should be considered in industrial applications depending on hydrogen purity requirements and economic feasibility.

## Graphical abstract

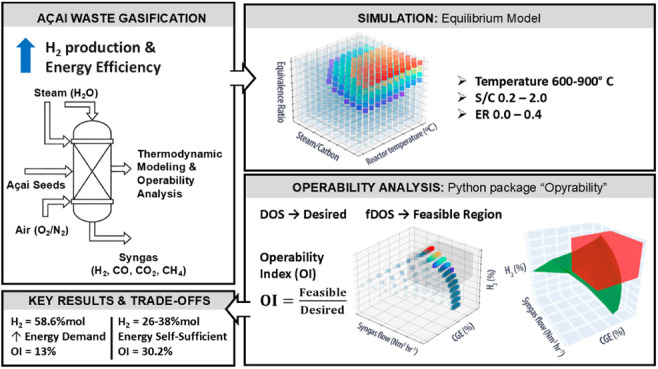

## 1 Introduction

Gasification is a well-established thermochemical process that converts carbon-based feedstocks, such as biomass, coal, and waste, into synthesis gas at high temperatures (typically 600 to 1000 °C) under substoichiometric conditions, using gasifying agents such as air, O_2_, CO_2_, and steam.^1,2^ The process produces syngas, mainly composed of H_2_, CO, CO_2_, and CH_4_, which can be used for power generation or as a feedstock for fuels and chemicals, along with byproducts such as char, tar, and ash.^3,4^

Biomass gasification involves a complex network of homogeneous and heterogeneous reactions,^3^ and its performance depends on both the physicochemical properties of the feedstock and the operating conditions. Biomass composition plays an important role, where higher cellulose and hemicellulose contents tend to favor syngas production, while optimal moisture content (10–20%) and low ash content (<2%) contribute to improved process efficiency and reduced tar formation.^1^ In addition, key operating parameters such as temperature, gasifying agent, and equivalence ratio (ER) strongly influence process performance. Higher temperatures promote tar cracking, steam addition enhances hydrogen production, and increasing ER reduces tar formation. However, it may shift the process toward partial combustion, thereby decreasing syngas quality and overall efficiency.^5^

The evaluation of biomass gasification systems can be effectively performed using computer simulations and process systems analysis, which provide insights into optimal operating conditions and energy requirements. These simulations rely on mathematical models generally classified into thermodynamic equilibrium and kinetic approaches.^6^ Kinetic models can be developed with varying levels of complexity, depending on the required level of detail. Njuguna et al.^7^ analyzed macadamia shell gasification using a kinetic model implemented in Aspen Plus that represents pyrolysis, combustion, and reduction zones and predicts char and tar. Tulu et al.^8^ developed a transient two-phase kinetic model in MATLAB that incorporates hydrodynamics, mass, and energy balances, including tar-cracking reactions. Rabaçal et al.^9^ applied computational fluid dynamics (CFD) using large eddy simulation (LES) with Lagrangian particle tracking and kinetic submodels for devolatilization and char combustion. Hasse et al.^10^ reviewed CFD approaches, such as LES, RANS, and DNS, coupled with advanced kinetic models, including CPD, FG-DVC, and FLASHCHAIN. Yu and Smith^11^ reported improved prediction of syngas composition and tar formation, while the inclusion of tar-related reactions enhances the representation of conversion pathways.^7,8^

The studies mentioned above demonstrate the capability of kinetic models to represent different reactor configurations and operating conditions. These models explicitly account for reaction mechanisms and rates, enabling detailed predictions of syngas composition and spatial and temporal variations. They are particularly suitable for nonequilibrium conditions, as they incorporate reactor configuration and hydrodynamic effects. However, these models require significant computational effort and reliable kinetic parameters, often demanding careful calibration.^12–14^

In contrast, thermodynamic equilibrium models assume that the reacting system reaches chemical equilibrium, typically determined through Gibbs free energy minimization, and are widely used in process simulators such as Aspen Plus for steady-state analysis.^11,14^ These models do not capture dynamic phenomena such as temperature and velocity gradients, which may lead to deviations under nonequilibrium conditions. Nevertheless, their ease of implementation, low computational cost, and independence from kinetic data make them suitable for preliminary assessments, process screening, and optimization studies.^14,15^ Despite their limitations, thermodynamic models have shown consistent performance in biomass gasification studies, supporting process analysis and optimization.

Zaman et al.^16^ employed a thermodynamic equilibrium model based on Gibbs free energy minimization using the RGIBBS block in Aspen Plus to simulate biomass gasification. This approach is widely recognized for its simplicity and for its ability to represent process behavior under steady-state conditions, with good agreement with experimental data. Similarly, Fu et al.^14^ simulated biomass gasification in an autothermal gasifier using an RGIBBS reactor, assuming isothermal, steady-state conditions and including the main gasification reactions. Model validation was performed by analyzing the effects of equivalence ratio (ER), temperature, and pressure on syngas composition and higher heating value (HHV). In addition, Okolie et al.^17^ applied thermodynamic equilibrium modeling to simulate the hydrothermal gasification of soybean and flax straw, comparing experimental results with theoretical predictions to evaluate deviations in hydrogen yield. Their analysis indicated that catalysts, such as KOH, yielded results closer to equilibrium conditions, supporting the applicability of thermodynamic models for optimization and scale-up studies.

Since operating conditions strongly influence gasification performance and, consequently, syngas quality, the use of process systems tools to identify optimal operating regions becomes essential, particularly when aiming to maximize the energetic potential of the produced gas. Among these tools, operability analysis provides a systematic framework that integrates design and control objectives from the early stages of process development. This approach evaluates a system’s ability to achieve desired outputs, considering the admissible ranges of input variables and the presence of disturbances. Based on the definition of operational sets and metrics such as the Operability Index (OI), it enables the assessment of process feasibility and performance^18–21^

In this context, this study focuses on the modeling and simulation of biomass gasification using a thermodynamic approach applied to açaí (*Euterpe oleracea*) pit waste, combined with operability analysis to optimize hydrogen composition, cold gas efficiency, and syngas flow rate. The analysis considers the steam-to-biomass ratio, air-to-carbon ratio, and reactor temperature as input variables. The selection of açaí pits is motivated by their high availability: Brazil produced approximately 1.95 million tons of açaí fruit in 2022, of which about 70% correspond to pits, resulting in approximately 1.37 million tons of residue.^22^ This abundance, combined with the need for proper waste management, makes açaí pits a promising feedstock for thermochemical conversion. The application of process systems and operability analysis to the gasification of açaí pits has not been reported in the literature, representing a relevant gap addressed in this study.

## 2 Materials and Methods

### 2.1 Characterization of Açaí Seed Biomass

The açaí seed residue was collected after pulp extraction and subjected to oven drying at 50 °C for 48 h until a constant mass was reached, ensuring adequate removal of surface moisture. Subsequently, the dried material was milled to the specified particle size of 60 mesh (approximately 250 μm) and stored for further characterization. Elemental analysis was performed using a FLASH 2000 CHNS/O Analyzer (Thermo Fisher) to determine the contents of carbon, hydrogen, nitrogen, sulfur, and oxygen. Proximate analysis for determining moisture, volatile matter, ash, and fixed carbon contents was carried out using a thermogravimetric analyzer (STA-6000, PerkinElmer) following ASTM standards E871-82 and E1755-0.^23–25^

### 2.2 Validation of the Mathematical Model

The validation of the proposed model was carried out using data from the literature under different operating conditions. In the first case, experimental data reported by Loha et al.^26^ were used, corresponding to the hydrothermal gasification of rice husk at a fixed temperature of 750 °C and varying steam-to-biomass mass ratios (S/B = 0.6, 1.0, and 1.32).

In addition, validation was also performed using data reported by Zaman et al.^16^ corresponding to rice husk gasification at a fixed steam-to-biomass ratio (S/B = 1) and temperatures ranging from 650 to 900 °C, allowing the evaluation of model predictions under different temperature conditions.

As a quantitative validation metric, the Root Mean Square Error (RMSE) was calculated between the values obtained from each model. This procedure allowed the evaluation of the agreement between the simulated results from both approaches and verified the consistency of the proposed model with respect to a well-established reference in the literature.

### 2.3 Mathematical Modeling of Gasification via Thermodynamic Approach

The mathematical model of the gasification process was developed in Aspen Plus V11 using a thermodynamic equilibrium-based approach. In the proposed model, the gasification products were assumed to be a mixture of H_2_O, N_2_, H_2_, CO_2_, CH_4_, and CO, while the char was assumed to be composed of solid carbon (C) and ash. Formation and decomposition of tar were not considered in the model, which, in general, may lead to an overestimation of the syngas components such as CO, CO_2_, and H_2_. Since thermal cracking of heavy hydrocarbons and conversion of tar into lighter gaseous species are favored by high gasification temperatures and by using steam as gasifying agent, respectively, only a slight deviation is to be expected by neglecting tar formation in the present work. The Peng–Robinson equation of state (PR) was chosen to adequately describe the behavior of gases and gas mixtures involved in the gasification process.

The thermodynamic properties of the chemical species were retrieved from the predefined databases of Aspen Plus. Both mixed-phase and solid-phase components were enabled to participate in chemical and phase equilibrium calculations, with temperature and pressure properties automatically updated by the software during simulations. The biomass was defined as a nonconventional component in the simulation. For this nonconventional component, the elemental composition data of the açaí seed was provided.

The process flow diagram of the gasification model developed in Aspen Plus is shown in Figure 1. The flowsheet represents a typical gasification configuration reported in the literature.^16^ in which the gasifier is modeled using two reactors: a yield reactor (“RYIELD”) and a Gibbs reactor (“RGIBBS”). However, additional unit blocks were included to provide a more realistic representation of the actual process, such as a stoichiometric reactor (“RSTOIC”), heat exchangers (“HEATER”, “HEATERX”, and “COOLER”), and a solid separator (“CYC”). This configuration, illustrated in Figure 1, allows for a more accurate assessment of the effects of input variables, such as the combustion zone temperature, steam-to-biomass ratio, and equivalence ratio on the process energy demand and the properties of the produced gas (e.g., hydrogen concentration in the syngas, cold gas efficiency, and syngas flow rate).

**Figure 1:**
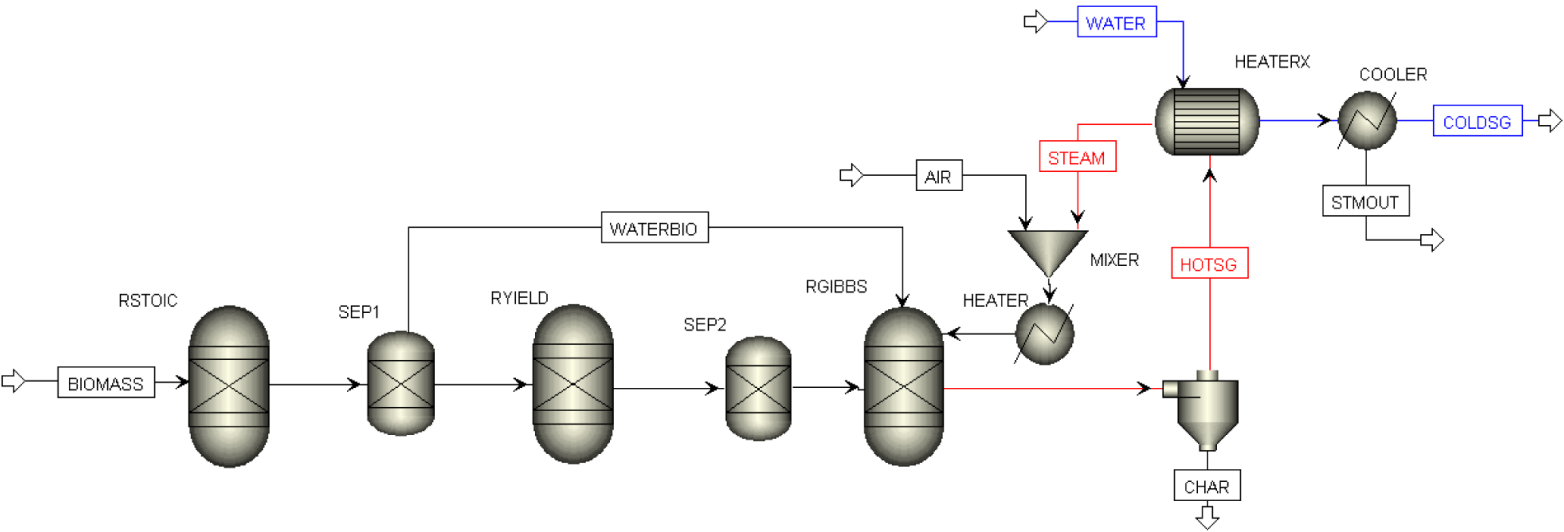
Process flowsheet of biomass gasification modeled in Aspen Plus; – hot streams; – cold streams.

The adopted gasification model assumes that the biomass is initially fed into a stoichiometric reactor, where the drying step is simulated. The water vapor released during this stage is routed through a bypass line to the Gibbs reactor, a strategy intended to adequately represent the drying process. After drying, the biomass is directed to the yield reactor, where the nonconventional biomass decomposes into a set of conventional components (C, O_2_, H_2_, N_2_, S). These components are then fed into the Gibbs reactor, for which the chemical reactions are listed in Table S1.

The gaseous product generated in the Gibbs reactor is directed to a solid separator, where solid particles are removed, and the upward gas stream proceeds to a shell-and-tube heat exchanger. This unit preheats the utility water entering the system while simultaneously cooling the gas. The operating condition establishes that the temperature difference between the gas and the water must not exceed 50 °C. Subsequently, the cooled gas is directed to a cooler to reduce the syngas temperature to ambient conditions (25 °C) and remove excess moisture. The steam stream generated during the process is then sent to a mixer, where it is combined with the air entering the system; both streams are heated in a heater to the operational temperature required by the Gibbs reactor and then directed into it. All operational conditions of this model are summarized in Table 1.

**Table 1:** Input Parameters for Simulation of the Biomass Gasification

Block/Stream	Parameter	Value	Unit
Biomass	Temperature	25	°C
	Mass flow	100	kg h^–1^
Water	Temperature	25	°C
	Steam/Biomass	0.2 to 2.0	-
Air	Temperature	25	°C
	Equivalence ratio	0.0 to 0.4	-
RGIBBS	Temperature	600 to 900	°C
RYIELD	Temperature	400	°C
	Efficiency	1	-
Separators	Efficiency	1	-

The calorific value of both the biomass and the syngas was evaluated; however, the software used does not provide metrics such as the lower heating value (LHV) and higher heating value (HHV) for the biomass feedstock. The LHV of the biomass was thus calculated using eq 1, known as the Strache–Lant equation, as described by Hosokai et al.^27^

1 $${\mathrm{LHV}}_{m}\left(\mathrm{MJ}/\mathrm{kg}\right)=34.1X_{C}+121.4X_{H}-15.3X_{O}+10.5X_{s}$$

where *X_i_* is the mass fraction of component *i* in the biomass.

The lower heating values (LHV) of the syngas were estimated by summing the individual heating values of its components, using the data reported by Waldheim et al.^28^ The LHV of the synthesis gas was calculated using eq 2 and crosschecked with the Aspen Plus stream property tool. There was almost no deviation between the two calculation methods, as can be seen in the comparison table presented in the Supporting Information, Table S.2.

2 $${\mathrm{LHV}}_{\mathrm{sg}}\left(\mathrm{MJ}/{\mathrm{N m}}^{3}\right)=10.78Y_{H_{2}}12.63Y_{\mathrm{CO}}+35.88Y_{{\mathrm{CH}}_{4}}$$

where *Y_i_* is the volumetric fraction of component *i* in the syngas. The cold gas efficiency (CGE) was calculated as described by Okolie et al.^17^ and is defined as the ratio between the energy contained in the produced syngas and the energy available in the biomass, as shown in eq 3.

3 $$\mathrm{CGE}\left(\%\right)=\frac{\phi_{\mathrm{sg}}\rho_{\mathrm{sg}}{\mathrm{LHV}}_{\mathrm{sg}}}{{ṁ}_{\mathrm{bm}}{\mathrm{LHV}}_{\mathrm{bm}}}\cdot 100$$

where *ρ*_sg_ represents the density of the synthesis gas, *ϕ*_sg_ corresponds to the volumetric flow rate of the synthesis gas, and *ṁ*_bm_denotes the mass flow rate of the biomass fed into the system. The equivalence ratio (ER) is defined as the ratio between the actual amount of air supplied to the process and the stoichiometric amount required for the complete combustion of the biomass, as described by Jahromi et al.,^29^ and is expressed in eq 4:

4 $$\mathrm{ER}=\frac{\mathrm{Actual}\;\mathrm{oxygen}\;\mathrm{flow}\;\mathrm{rate}}{\mathrm{ideal}\;\mathrm{oxygen}\;\mathrm{flow}\;\mathrm{rate}-\mathrm{biomass}\;\mathrm{oxygen}}$$

The equations for the metrics are not natively implemented in the simulation software, requiring them to be programmed in a Fortran subroutine to enable their calculations using Aspen information. These metrics were applied to support the optimization of the gasification process, and the implementation of which is discussed in the following section.

### 2.4 Optimization through Operability Analysis

The operability analysis was applied to the gasification process through a computational code developed in Python, based on the open-source tool known as Opyrability.^19^ The optimization aimed to identify the operational regions that simultaneously maximize outputs variables such as molar fraction of hydrogen in the syngas, the volumetric flow rate of the syngas, and the CGE. To achieve this, the steady-state gasification model, based on a thermodynamic approach, was represented by a system model (*M*) composed of mass and energy balances, with the reactor temperature (*T*), the steam-to-biomass mass ratio (θ_s_), and the air-to-carbon mass ratio (θ_a_) as input variables. The molar fraction of water in the produced syngas (*γ*_*w,out*_) was constrained to a maximum of 3.5% (mol/mol), as expressed in eq 5.

5 $$M=\{\begin{array}{l} ṁ=f\left(T,\theta_{s},\theta_{a}\right)\\ Ė=g\left(T,\theta_{s},\theta_{a}\right)\\ \gamma_{w,out}\leq 0.035 \end{array}$$

A mapping of the model was performed to assess how the input variables affect the gasification process. The mapping establishes the relationship between the set of accessible operational inputs (AIS – Achievable Input Set) and the set of outputs that be achieved by the process (AOS – Achievable Output Set), allowing the identification of system responses for different operational configurations. The procedure consists of systematically exploring the space of control/input variables defined by the AIS, using the process’s thermodynamic model to simulate the output variables behavior. In this way, the impact of each operational condition on the gasification products is evaluated, enabling the identification of operating regions that meet the desired objectives, such as the maximization of hydrogen production or the minimization of energy demand.

The AIS set was defined considering three main operational variables: (i) the combustion reactor temperature, ranging from 600 to 900 °C; (ii) the steam-to-biomass mass ratio (S/B), ranging from 0.2 to 2, previously adjusted to discount the intrinsic moisture of the biomass; and (iii) the equivalence ratio (ER), ranging from 0 to 0.4. These intervals were selected based on ranges reported in the biomass gasification literature.^29^ The S/B ratio influences reforming and water–gas shift reactions and therefore affects hydrogen formation and gas composition. Moderate S/B values are generally preferred because increasing steam promotes hydrogen production, whereas excessive steam may reduce the heating value of the syngas and increase the energy demand of the process.^13,30^ The mathematical formalization of these sets is presented in eqs 6 and 7.

6 $$\mathrm{AIS}=\left\{u_{i,j,k}\in R^{3}|\left(600,0.2,0\right)\leq u_{i,j,k}\leq \left(900,2,0.4\right)\right\}$$

7 $$\mathrm{AOS}=\left\{y_{i,j,k}\in R^{3}|y_{i,j,k}=M\left(u_{i,j,k}\right)\;\mathrm{and}\;u_{i,j,k}\in \mathrm{AIS}\right\}$$

By mapping these variables, it becomes possible to identify the system’s ability to adjust its inputs to reach the Desired Output Set (DOS), which represents the performance targets established for the process. To ensure that the optimization of the desired variables is carried out consistently, the DOS ranges were constructed based on the maximum capacity of the process, as determined by the AOS. Thus, the output variable ranges such as the hydrogen concentration in the syngas $\left(\gamma_{H_{2},out}\right)$ , the cold gas efficiency (CGE), and the volumetric flow rate of syngas (ϕ_sg_) were defined within intervals ranging from 70% of their maximum achievable value to their maximum values themselves. This was evaluated under two conditions: one in which there is no limitation on energy demand (*Q*_*d*_), and another in which the energy demand must be equal to or less than zero. The definitions of these intervals are represented by the sets of eqs 8 and 9:

8 $${\mathrm{DOS}}_{1}=\left\{y_{i,j,k}\in R^{3}|0.70\left(\mathrm{CGE},\phi_{\mathrm{sg}},\gamma_{H_{2},out}\right)\leq y_{i,j,k}\leq {\left(\mathrm{CGE},\phi_{\mathrm{sg}},\gamma_{H_{2},out}\right)|}_{\max}\right\}$$

9 $${\mathrm{DOS}}_{2}=\left\{y_{i,j,k}\in R^{3}{\left|0.70\left(\mathrm{CGE},\phi_{\mathrm{sg}},\gamma_{H_{2},out}\right)\right|}_{Q_{d}<0}\leq y_{i,j,k}\leq {\left(\mathrm{CGE},\phi_{\mathrm{sg}},\gamma_{H_{2},out}\right)|}_{Q_{d}<0}\right\}$$

The Desired Input Set (DIS) was obtained through inverse mapping using a Nonlinear Programming (NLP)-based approach. The algorithm employed for the NLP was adapted from the Opyrability tool, with several modifications to allow gasification process simulation data as the starting point for minimization in each iteration and convergence boundaries limited to the AIS range. These adaptations aimed to enhance the accuracy of the model’s convergence. The minimization method used was the Nelder–Mead Simplex algorithm.^31^ The mathematical definition of the DIS set are represented in eq 10 below:

10 $${\mathrm{DIS}}_{i}=\left\{u_{i,j,k}\in R^{3}|M^{-1}\left(y_{i,j,k}\right)\;\mathrm{and}{\;y}_{i,j,k}\in {\mathrm{DOS}}_{i}\right\}$$

The points within the DOS volume that result from inputs in the DIS set are referred to as the feasible Desired Output Set (fDOS). The Operability Index (OI) was defined as a key metric for evaluating the operability of processes, such as gasification. This metric is used to quantify the system’s ability to achieve the desired outputs within the operational limits.^20^ For this purpose, the AOS and DOS intervals are represented as polytopes with volume (μ), enabling a geometric interpretation of these sets. Accordingly, the OI is defined as the ratio between the intersection of achievable and desired, *μ*(AOS ∩ DOS), with the desired, *μ*(DOS), as shown in eq 11:

11 $$\mathrm{OI}=\frac{\mu \left(\mathrm{AOS}\cap \mathrm{DOS}\right)}{\mu \left(\mathrm{DOS}\right)}\cdot 100$$

## 3 Results and Discussion

### 3.1 Model Validation

Table 2 presents the dry molar composition of syngas obtained from the gasification of rice husk under two different validation conditions. The first data set corresponds to a fixed temperature of 750 °C and varying steam-to-biomass mass ratios (S/B = 0.6, 1.0, and 1.32), using experimental data reported by Loha et al.^26^ and Zaman et al.^16^ The second data set corresponds to a fixed steam-to-biomass ratio (S/B = 1) and temperatures ranging from 650 to 900 °C, based on simulation results reported by Zaman et al.^16^

**Table 2:** Comparison of Dry Basis Molar Composition (Mol %) of Syngas for Rice Husk Gasification Under Two Validation Conditions: (i) Experimental Data at 750 °C with Varying Steam-To-Biomass Ratios and (ii) Data at S/B = 1 with Temperatures Ranging from 650 to 900 °C

S/B effect at 750 °C									
	Experimental data from Loha et al.^26^				Proposed model (this work)				
S/B	H_2_	CO	CO_2_	CH_4_	H_2_	CO	CO_2_	CH_4_	RMSE
0.6	48.8	27.5	19.5	4.2	51.08	33.07	15.49	0.35	4.10
1	49.5	23.7	21.2	5.6	55.47	22.07	22.37	0.09	4.18
1.32	52.3	17.75	22.25	7.4	57.26	17.33	25.36	0.03	4.71

Temperature effect at S/B = 1									
	Data from and Zaman et al.^16^				Proposed model (this work)				
*T* (°C)	H_2_	CO	CO_2_	CH_4_	H_2_	CO	CO_2_	CH_4_	RMSE
650	55.27	17.23	25.80	1.34	55.40	17.30	26.00	1.30	0.12
700	55.71	20.00	23.84	0.27	55.90	20.00	23.80	0.30	0.09
750	55.36	22.23	22.32	0.00	55.50	22.10	22.40	0.10	0.11
800	54.64	24.02	20.98	0.00	54.90	23.90	21.20	0.00	0.18
850	54.20	25.45	20.09	0.00	54.30	25.50	20.10	0.00	0.06
900	53.66	26.88	19.02	0.00	53.80	27.00	19.20	0.00	0.16

In the first case (fixed temperature), it can be observed that the molar concentration of hydrogen increases with the steam-to-biomass ratio, which is consistent with the promotion of the water–gas shift reaction (see Table S1). As the steam content increases, this reaction is shifted toward the formation of hydrogen and carbon dioxide, leading to a simultaneous increase in both components. Consequently, carbon monoxide concentration decreases, since it is consumed as a reactant in the water–gas shift reaction. This coupled behavior explains the observed trends of increasing H_2_ and CO_2_ and decreasing CO with higher steam-to-biomass ratios.

The values predicted by the model were compared with experimental data reported by Loha et al.,^26^ which are also presented by Zaman et al.^16^ under the same operating conditions. The slight deviations observed between simulated and experimental values, reflected by root-mean-square error (RMSE) values below 5.0, may be attributed to the use of a thermodynamic equilibrium model in which tar formation is not considered. As a result, intermediate carbon-containing species, including methane, are assumed to be preferentially converted into permanent gases, which can lead to a slight overestimation of components such as H_2_, CO, and CO_2_.

However, equilibrium-based gasification models commonly neglect tar formation in process feasibility and optimization studies, since its concentration strongly depends on operating conditions such as temperature.^26,32^ High gasification temperatures promote the thermal cracking of heavy hydrocarbons, while the presence of steam enhances reforming reactions that convert tar into lighter gaseous species.^26^ Under these conditions, tar levels tend to be significantly reduced compared to the main syngas components, and its exclusion does not significantly affect the predictive capability of the model for the conditions evaluated in this work. Furthermore, the present model focuses on the main syngas components, which are the primary interest for the evaluation of the gasification process.

The second validation case, presented in Table 2, evaluates the ability of the model to reproduce the variation of syngas composition as a function of temperature at a fixed steam-to-biomass ratio (S/B = 1), based on the results reported by Zaman et al.^16^ The comparison shows a strong agreement between the proposed model and the literature data across the entire temperature range (650–900 °C), with low RMSE values (<0.2) for all evaluated conditions. These results are also illustrated in Figure 2, where the continuous curves represent the model predictions and the discrete points correspond to the data reported in the literature.

**Figure 2:**
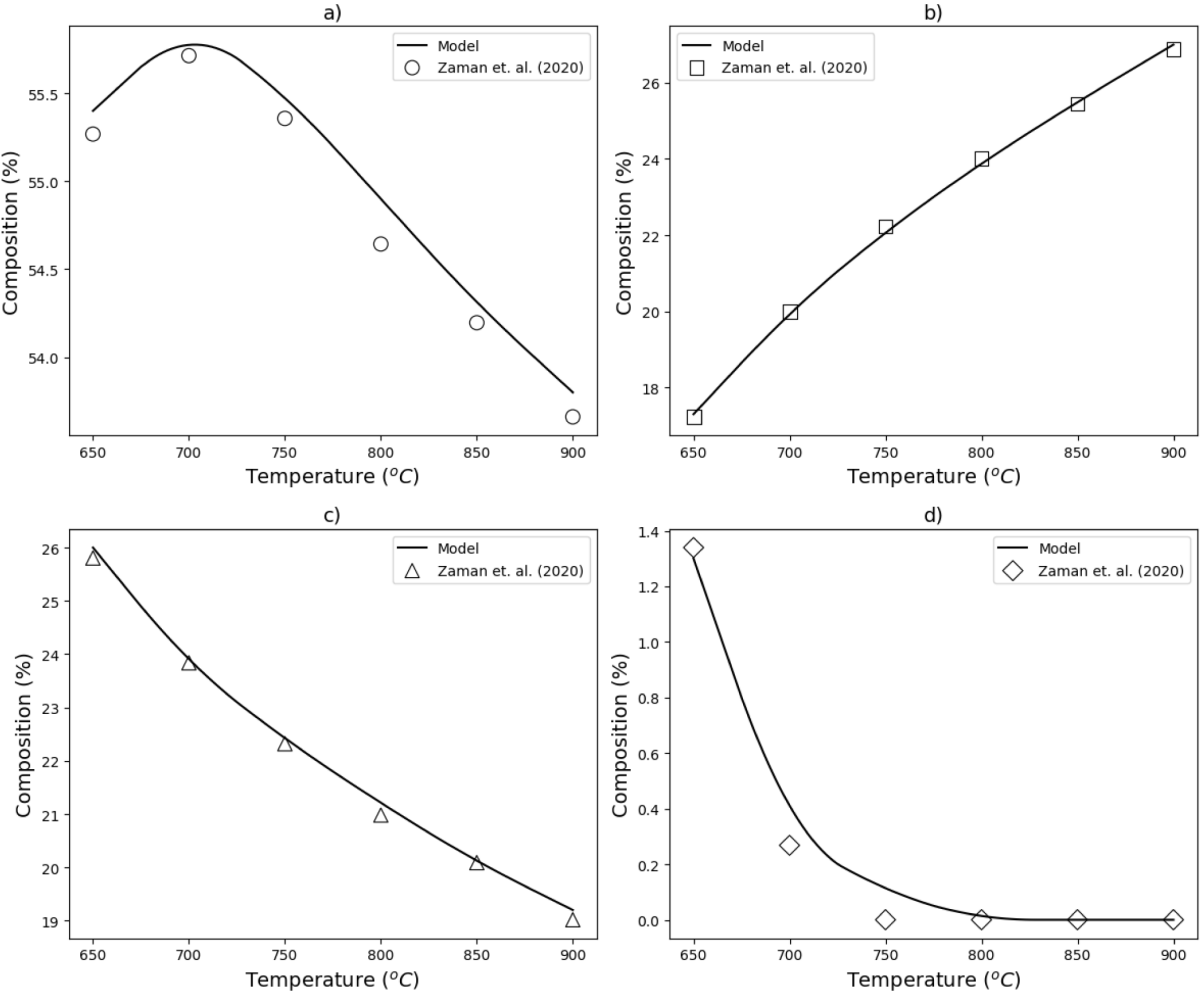
Dry basis composition of syngas produced from rice husk gasification at different temperatures (650 to 900 °C) and a steam-to-biomass mass ratio of 1:1. Data from the literature (points) and simulation results from the proposed model (lines): a) H_2_, b) CO_2_, c) CO, and d) CH_4_.

From a compositional standpoint, the model accurately captures the expected thermodynamic trends associated with increasing temperature. A slight decrease in hydrogen concentration is observed, accompanied by an increase in carbon monoxide and a reduction in carbon dioxide, which is consistent with the equilibrium shift of the water–gas shift reaction toward CO formation at higher temperatures. Overall, the comparison indicates good agreement with results reported in the literature, demonstrating that the proposed model is consistent and representative of the system behavior under the evaluated conditions.

### 3.2 Elemental and Proximate Analysis of Açaí Seed Biomass

The results of the elemental and proximate analyses of the açaí seed biomass are presented in Table 3 The elemental composition is consistent with that of other lignocellulosic biomasses reported in the literature, such as the rice husk modeled by Zaman et al.,^16^ and the soybean straw and flax straw studied by Okolie et al.,^17^ comparing the values at dry and ash-free basis.

**Table 3:** Proximate and Ultimate Analysis of the Açaí Seed Biomass^a^

Proximate analysis (wt %)		Ultimate analysis (wt %)	
Moisture	8.3 ± 0.32	C	47.7 ± 0.18
Fixed carbon	19.2 ± 0.85	H	6.9 ± 0.19
Volatile material	70.4 ± 0.20	N	0.8 ± 0.03
Ash	2.0 ± 0.18	O	44.6 ± 0.15

a The estimated lower heating value (LHV) is 18.1 MJ/kg.

Regarding the proximate analysis, the results obtained in the present work are similar to those of flax straw reported by Okolie et al.^17^ The moisture content of 8.3% suggests a relatively dry biomass, which is a promising result for conventional energy conversion processes since high moisture content can reduce process efficiency. The high volatile matter fraction of 70.4% indicates a strong potential for gas release during the pyrolysis stage, while the low ash content (2.0%) suggests that the biomass contains few mineral impurities, which is advantageous in avoiding operational problems in thermochemical reactors, such as fouling and slag formation. Moreover, the fixed carbon content was 19.2%, reflecting the fraction of the material that remains after volatile release and contributes to the formation of residual char.

The elemental analysis revealed a composition rich in carbon (47.7%) and oxygen (44.58%), with hydrogen and nitrogen contents of 6.95% and 0.8%, respectively. The absence of chlorine and sulfur in the sample is a positive aspect, as it prevents environmental issues related to the emission of pollutant compounds such as SOx and HCl during the thermochemical conversion of biomass. The results reinforce the potential of açaí seed biomass as a viable energy feedstock, especially for gasification processes, due to its high volatile matter content and low ash content.

### 3.3 Effects of Air Feeding on the Gasification of Açaí Seed Biomass

The influence of the equivalence ratio (ER) on the composition of the synthesis gas produced during gasification was analyzed at a combustion zone temperature of 690 °C without steam feeding, and the variation in the composition (mol %) of the main gaseous components (H_2_, CO, CO_2_, CH_4_, H_2_O, and N_2_) as a function of ER is presented in Figure 3. In Figure 3a, it can be observed that increasing ER leads to a decrease in the concentrations of carbon monoxide (CO) and molecular hydrogen (H_2_). This trend can be attributed to the higher oxygen supply to the system, which favors the oxidation of CO to carbon dioxide (CO_2_) and reduces the extent of reforming and water–gas shift reactions that contribute to H_2_ generation. As a result, the CO_2_ produced in the combustion zone exceeds the conversion capacity of the reduction bed, indicating intensification of the oxidation reactions.^32^

**Figure 3:**
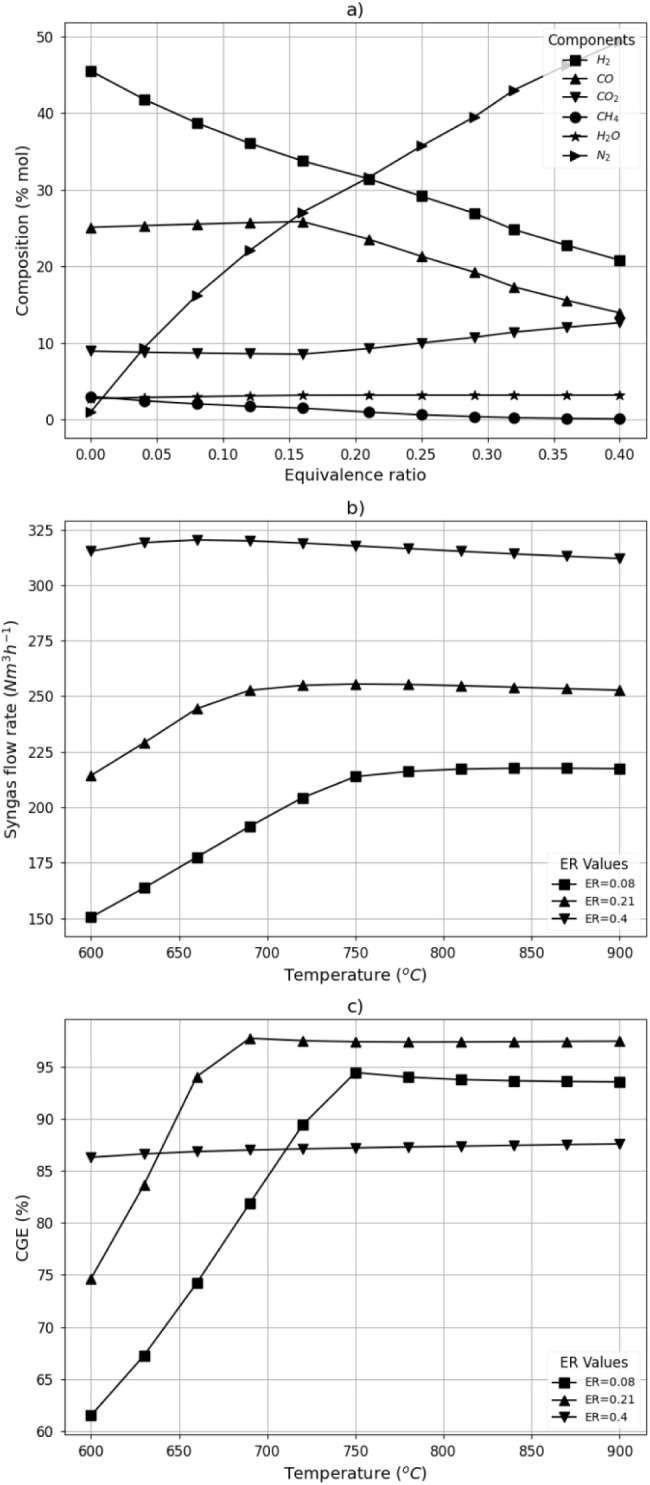
a) Syngas composition as a function of equivalence ratio (ER) at a combustion temperature of 690 °C and S/B = 0.20; b) Syngas flow rate profile over the combustion temperature range of 600–900 °C and ER range of 0.08–0.4; c) Cold gas efficiency (CGE) of the syngas under the same temperature and ER conditions.

The methane (CH_4_) content in the syngas also decreases with increasing ER, suggesting that a more oxidizing atmosphere inhibits methanation reactions and favors the conversion of CH_4_ into CO and H_2_ through dry reforming. Meanwhile, the water vapor (H_2_O) concentration remains practically constant due to the operation of the heat exchanger at the system outlet.

Furthermore, the nitrogen (N_2_) content increases significantly with increasing ER. This behavior is expected since a higher air supply (used as the oxidizing agent) results in greater dilution of the combustible gases due to the introduction of atmospheric nitrogen. Overall, the results indicate that a higher ER enhances combustion and promotes the oxidation of intermediate products, increasing the proportion of CO_2_ and N_2_ in the syngas while reducing the concentrations of CO, H_2_, and CH_4_.^32,33^

Figure 3b presents the flow rate profile of the synthesis gas as a function of the combustion zone temperature for three different ER values. For ER = 0.08, a linear increase in syngas volumetric flow is observed from 600 °C (≈150 N m^3^ h^–1^) to 750 °C (≈217 N m^3^ h^–1^), followed by stabilization up to 900 °C. A similar pattern is observed for ER = 0.2, with a linear increase from 600 °C (≈217 N m^3^ h^–1^) to ≈675 °C (≈250 N m^3^ h^–1^). This behavior suggests that, within a certain temperature range, the thermochemical conversion of biomass and syngas production reaches a limit imposed by the dominant reaction regime in the process. In contrast, the syngas flow rate for ER = 0.4 reaches a maximum point (≈320 N m^3^ h^–1^) at approximately 660 °C, followed by a slight decrease up to 900 °C (≈310 N m^3^ h^–1^). This can be attributed to increased direct oxidation of gaseous products (e.g., CH_4_, CO, H_2_) due to the higher availability of oxygen. As expected, the syngas flow rate increases with ER due to higher nitrogen injection into the system. There is also a tendency toward stabilization of the syngas flow rate under high ER conditions. This behavior is closely related to the intensification of exothermic reactions, the rapid conversion of biomass, and the limitations imposed by secondary oxidation reactions, which reduce the potential for increased gas volume with rising temperature.

Figure 3c shows the cold gas efficiency (CGE) profile as a function of combustion zone temperature, considering three different equivalence ratios (ER). A similar trend is observed for ER values of 0.08 and 0.2, with an almost linear increase in CGE across the temperature range from 61.5% to 91.5% for ER = 0.08 and from 74.0% to 97.7% for ER = 0.2. In both cases, stabilization of efficiency is observed from 750 and 690 °C onward, respectively. This behavior is consistent with the syngas composition, as high temperatures associated with oxygen-deficient atmospheres favor CO formation and reduce oxidative reactions, preserving the energy potential of components such as H_2_ and CH_4_. For the condition of ER = 0.4, CGE remains practically constant, with an average value around 87% throughout the evaluated temperature range (600 to 900 °C). At higher ER values, where greater conversion of CO and H_2_ into CO_2_ and H_2_O occurs, cold gas efficiency tends to decrease.

ER adjustment proves to be a critical parameter for optimizing the composition of the synthesis gas, whether for direct combustion or for chemical processes that require higher concentrations of CO and H_2_. The results also highlight that ER directly affects the amount of gas produced and that there is a temperature limit at which the gas flow reaches a maximum before stabilizing or decreasing. This information is essential for gasification process optimization, emphasizing that the ER variable must be carefully considered in operability-based optimization analysis.

### 3.4 Influence of Steam Input on the Gasification Process

Figure 4a shows the synthesis gas composition as a function of the steam-to-biomass ratio (S/B ratio) while keeping the combustion zone temperature and the equivalence ratio constant (690 °C and 0.08). These conditions were selected due to their association with greater energetic stability of the process, as energy demand remains low, approaching the autothermal condition. It is observed that, with increasing S/B ratio, the molar fraction of hydrogen (H_2_) progressively increases, from 38.7% to 49.7%. This behavior is attributed to the intensification of the water–gas shift reaction (CO + H_2_O ⇄ CO_2_ + H_2_), which favors hydrogen production.

**Figure 4:**
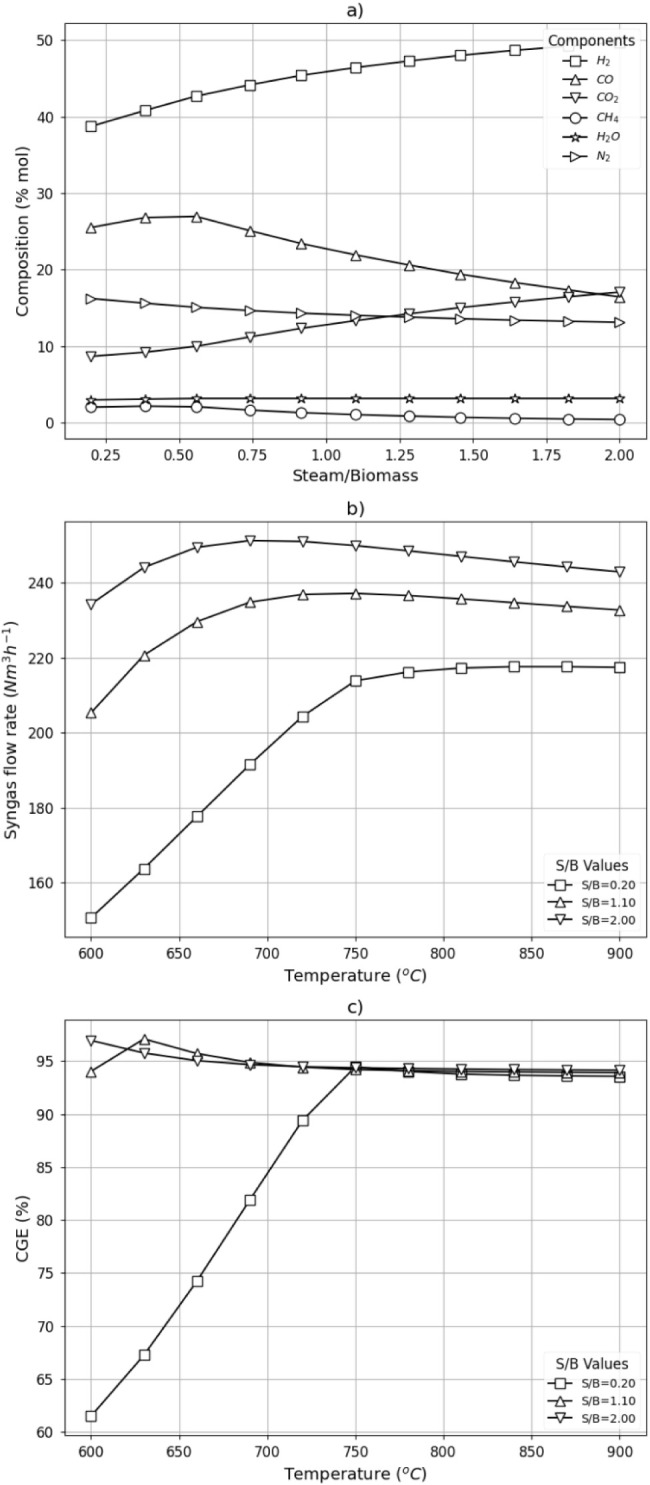
a) Syngas composition as a function of *Steam-to-biomass ratio* (S/B) to biomass gasification at 690 °C and ER of 0.08. b) Flow rate profile of the syngas produced at combustion temperature range of 600–900 °C, ER of 0.08, and S/B ratio of 0.2, 1.1, and 2.0. c) CGE of the syngas produced at temperature range of 600–900 °C, equivalence ratio of 0.08, and S/B ratio of 0.2, 1.1 and 2.0.

The concentration of carbon monoxide (CO) remains relatively stable up to an S/B ratio of approximately 0.6, after which it declines from 27.5% at S/B = 0.2 to about 16.5% at S/B = 2. This trend results from the interplay between the water–gas shift reaction, which consumes CO, and the steam methane reforming reaction (CH_4_ + H_2_O ⇄ CO + 3H_2_), which generates CO. At intermediate S/B ratios, these reactions are balanced, maintaining a nearly constant CO concentration. As methane is gradually depleted through reforming, its reduced availability limits further CO production, allowing CO consumption via the water–gas shift reaction to predominate and causing the observed decrease in CO concentration.

As shown in Figure 4a, the concentration of carbon dioxide (CO_2_), a product of the water–gas shift reaction, increases from 8.7% to 17.1%, while CO decreases from 26.8% to 16.5%. This behavior highlights the predominance of partial oxidation and shift reactions (conversion of CO to CO2) under the analyzed conditions. Methane (CH_4_) concentration progressively decreases over the examined range, from 2.1% to 0.4%, indicating a shift in the methanation reaction (CO + 3H_2_ ⇄ CH_4_ + H_2_O) in the reverse direction, favoring CH_4_ conversion into CO and H_2_ under conditions of greater steam availability. Due to condensation in the heat exchanger, the water vapor fraction (H_2_O) in the syngas remains practically constant around the saturation moisture level of 3.2%. Nitrogen (N_2_), in turn, decreases slightly from 16.2% to 13.1%, possibly due to gas dilution caused by increased production of gaseous species from reactions with steam.

In addition to the composition, the syngas volumetric flow rate is also influenced by the S/B ratio. As shown in Figure 4b, for S/B = 0.20, there is a linear increase in the syngas volumetric flow between 600 °C (≈150 N m^3^ h^–1^) and 750 °C (≈217 N m^3^ h^–1^), followed by stabilization up to 900 °C. For S/B ratios of 1.1 and 2.0, the flow increases up to a peak (≈237 N m^3^ h^–1^ for S/B = 1.1 and ≈243 N m^3^ h^–1^ for S/B = 2), followed by a slight decrease up to 900 °C. This behavior may be attributed to the increased formation of gaseous species, particularly H_2_ and CO_2_, resulting from steam-involved reactions. However, the reduction in flow at higher temperatures under elevated S/B conditions suggests that secondary reactions become more significant, leading to pathways in which the total number of moles in the gas phase is reduced. As a result, even with ongoing conversion, the overall volumetric flow tends to decrease.

Figure 4c presents the cold gas efficiency (CGE) profile of the syngas as a function of the combustion zone temperature for three different S/B conditions. For an S/B ratio of 0.20, a linear increase in CGE is observed (61.5% → 94%), followed by stabilization from 750 °C onward. This behavior may be related to the increase in hydrogen concentration, which contributes energetically to the syngas until the mechanisms responsible for its production reach near-equilibrium. In contrast, for S/B ratios of 1.1 and 2.0, this equilibrium is reached at lower temperatures.

The relationship between input and output variables such as temperature, equivalence ratio (ER), and steam-to-biomass ratio (S/B) exhibits nonlinear behavior, particularly regarding hydrogen production and cold gas efficiency. These findings reinforce that using optimization techniques based on operability analysis is particularly suitable for the gasification model studied. In this context, operability analysis enables the mapping of feasible and optimal operating regions that might be overlooked by traditional optimization methods based solely on mathematical modeling. By simultaneously considering multiple constraints and objectives, this approach provides a robust foundation for optimizing complex processes such as biomass gasification.

### 3.5 Operability Analysis of the Gasification Process

#### 3.5.1 Process Energy Demand: Without Constraint

The optimization of the gasification process was carried out through operability analysis, identifying feasible input points (in the DIS) and feasible desired output points (in the fDOS) that satisfy the desired operating conditions. Figure 5a shows the Available Input Set (AIS), with all combinations of input variables within the process operating domain represented in transparent blue.

**Figure 5:**
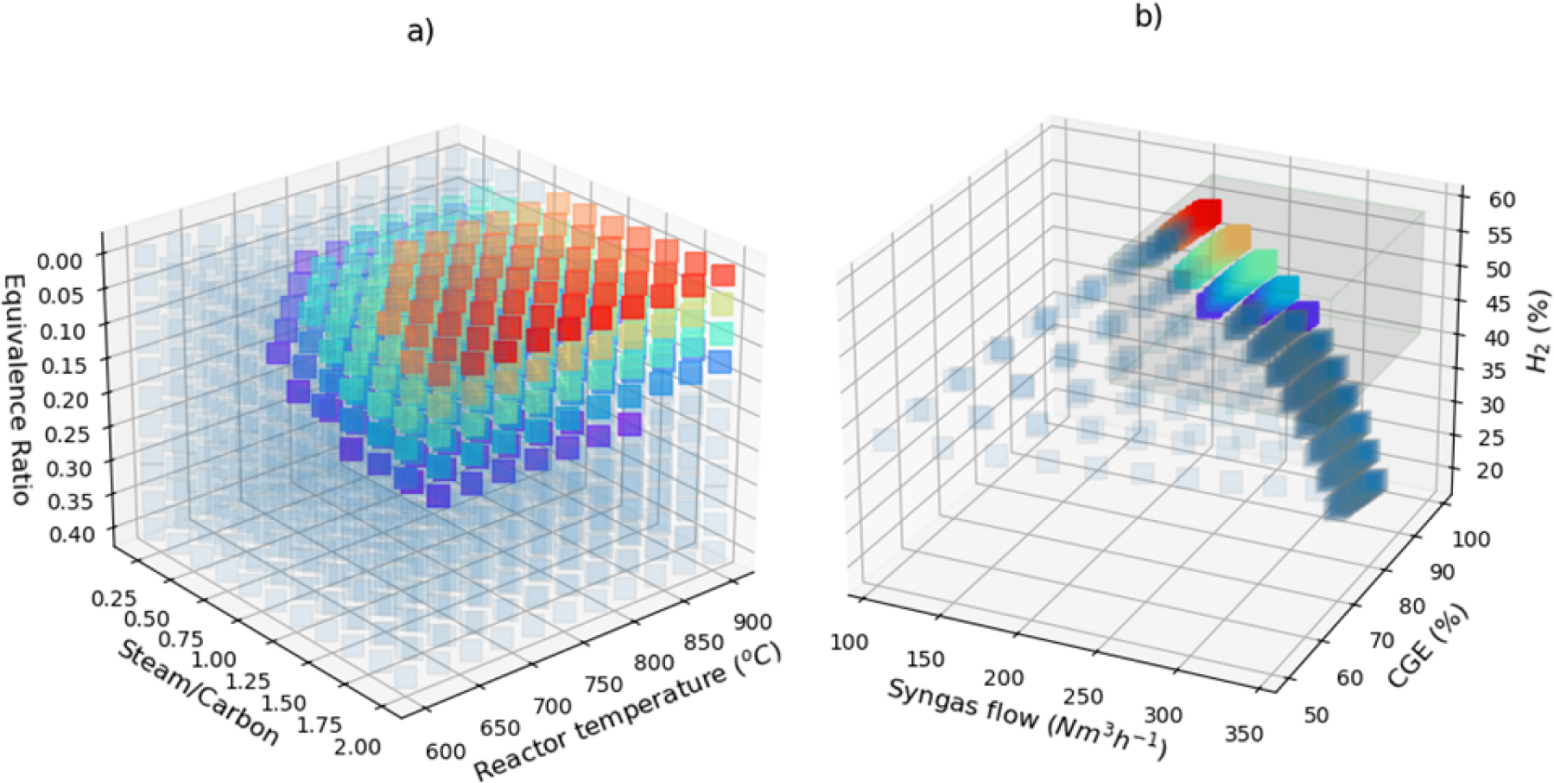
Operability analysis without energy demand constraint. a) Available Input Set (AIS), with feasible and desirable inputs (DIS) highlighted in color; b) Achievable Output Set (AOS), with the Desired Output Set (DOS) represented as a transparent gray polytope and fDOS points shown in corresponding colors.

Within this space, subset of input conditions, referred to as the Desired Input Set, was identified, where each colored point corresponds to an output point located in the desired region. These points were selected based on the simultaneous maximization of hydrogen production, syngas flow rate, and cold gas efficiency, without imposing any constraint on the process energy demand.

The operability analysis revealed that the operational conditions within the optimized region varied significantly and nonlinearly across the entire analyzed range of temperature and S/B. The ER varied between 0.0 and 0.16, indicating that adjustments to these variables must be precise and are crucial for achieving improved process performance.

Figure 5b presents the Achievable Output Set, where the translucent blue points represent all possible model responses for the different input points. Within this space, the optimal region was defined, represented by a transparent gray volume, which delineates the boundaries of ideal operating conditions. The colored points within this region correspond to the set of feasible and desirable outputs, with colors directly matching the corresponding points in the desired input set shown in the first graph (Figure 5). This relationship makes it possible to identify which operational conditions result in desirable values for syngas flow rate, cold gas efficiency, and hydrogen concentration.

In the optimized region, cold gas efficiency ranged from 85.8 to 98.5%, while the hydrogen content in the synthesis gas varied between 41.0 and 58.6 mol %. The syngas flow rate ranged from 207 to 275 N m^3^ h^–1^, while the energy demand varied between 78 and 444 MJ h^–1^. These values show that optimization was able to identify a set of operating conditions that maximize both efficiency and gas production, although without controlling energy demand.

The energy demand profile of the açaí seed gasification process, as a function of operational conditions (T, ER, S/B ratio), is shown in Figure 6. Energy demand is represented as a heat map applied to the desired input set, where the color variation indicates the magnitude of energy consumption as process heat duty. This visualization allows the distribution of energy consumption within the optimized region to be assessed, making it easier to identify conditions that offer a better balance between thermal efficiency and process performance.

**Figure 6:**
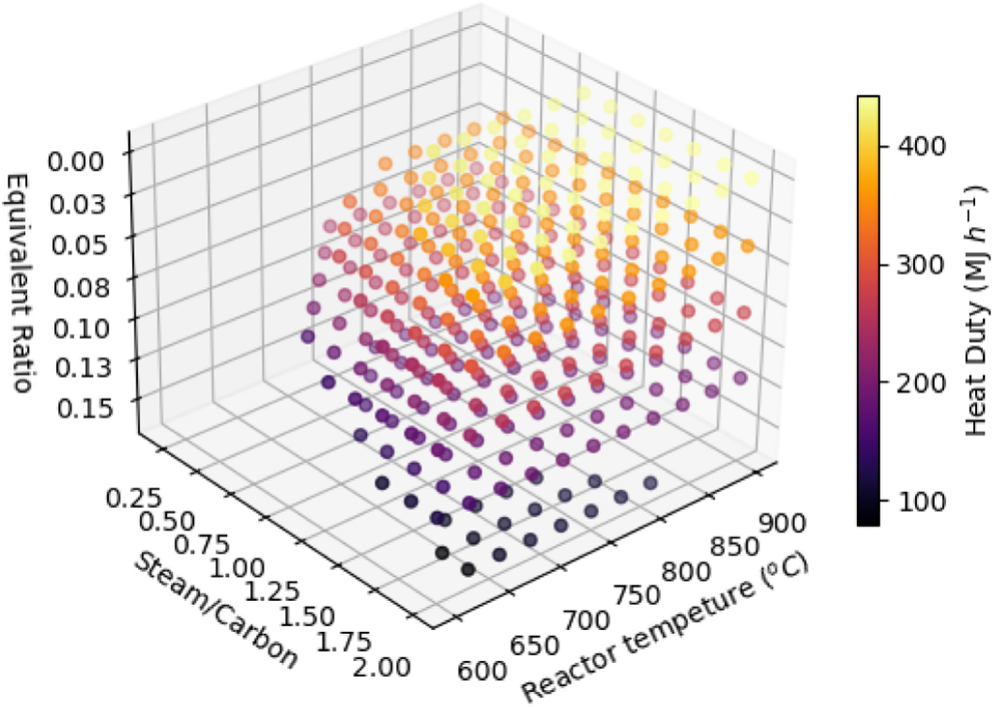
Energy demand profile of the açaí seed gasification process as a function of combustion temperature, equivalence ratio, and steam-to-biomass ratio. Operability analysis is performed without energy constraint, with heat duty mapped over the DIS region.

The relationship between input and output variables is not linear, particularly concerning hydrogen production and cold gas efficiency. This characteristic reinforces the importance of operability analysis, as it allows for mapping patterns and optimal regions that might not be evident through traditional optimization approaches based solely on conventional mathematical methods that calculate one optimal operating point, as opposed to entire geometric regions. In this context, the application of the methodology revealed that only 13% of the evaluated operating region corresponds to conditions that are both desirable and feasible, as indicated by the operability index calculated and shown in Figure 7.

**Figure 7:**
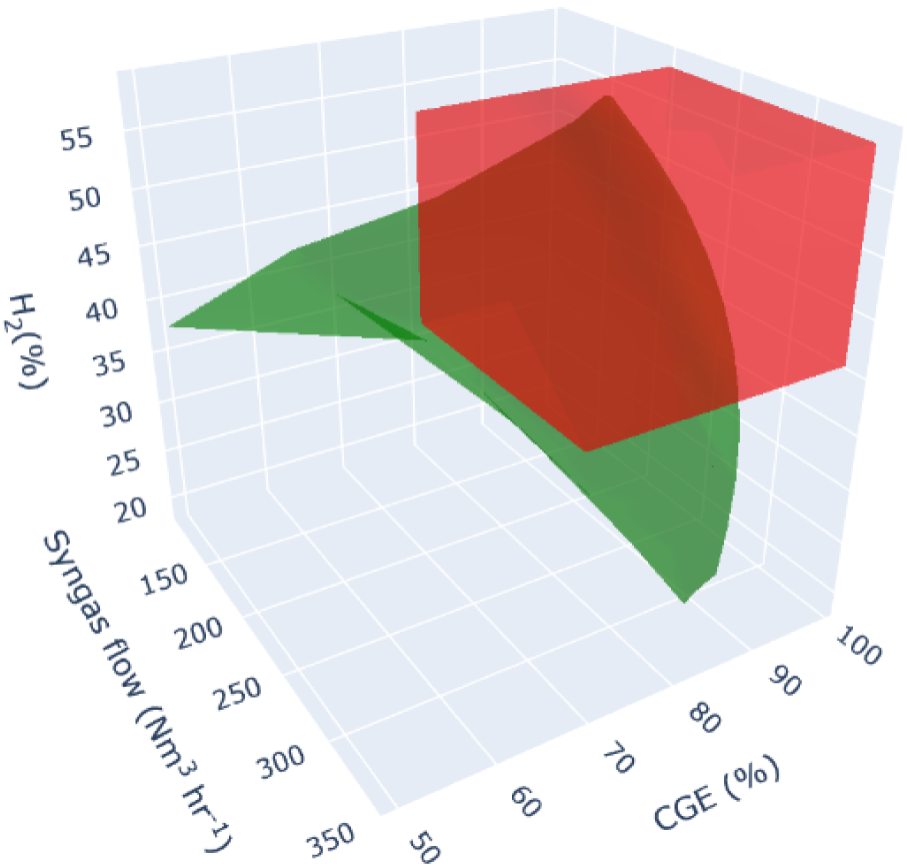
Operability map of the gasification process without energy constraint: intersection between AOS (green polytope) and DOS (red polytope). Operability index = 13%.

The graph shown in Figure 7 illustrates the operability analysis of the gasification process without energy constraints. In this figure, the green surface represents the polytope generated from the simulated process results, i.e., the set of all feasible system outputs within the explored sampling space. The red region defines the desired output set, representing the target performance conditions considered optimal or preferable for applying the synthesis gas.

The partial overlap between the surfaces indicates that only a fraction of the operating space can simultaneously meet the criteria of high efficiency, high gas flow, and hydrogen enrichment. Therefore, the graph demonstrates that, even in the absence of energy constraints, operational freedom is relatively limited, as evidenced by an operability index of only 13%. Nonetheless, it still allows for achieving high hydrogen concentrations, generally exceeding 50% of the molar composition of the synthesis gas.

#### 3.5.2 Process Energy Demand: With Constraint

The operability analysis of the gasification process under energy demand constraint followed the same procedures as in the previous case. However, the delimitation of the optimal output region (DOS) was performed, considering exclusively the points with negative energy demand (Heat Duty <0). This approach ensured that the process optimization occurred under energetically feasible conditions, resulting in an operability index of 30.2% as explained below, which indicates greater operability and a more efficient process under the energy criterion.

Figure 8a shows the profile of the desired input variables (DIS), revealing a wide variation across the entire temperature and S/B range. However, the fed ER exhibited a distinct interval, ranging from 0.16 to 0.35, in contrast to the previous case (without constraints). Figure 8b presents the set of AOS and fDOS output points, highlighting the optimal output region (DOS) with a transparent gray box. The results indicate that, within this optimized region, the cold gas efficiency (CGE) ranged from 79 to 98%, hydrogen concentration (H_2_) varied between 26 and 38% (v/v), and the syngas flow rate ranged from 207 to 332 N m^3^ h^–1^.

**Figure 8:**
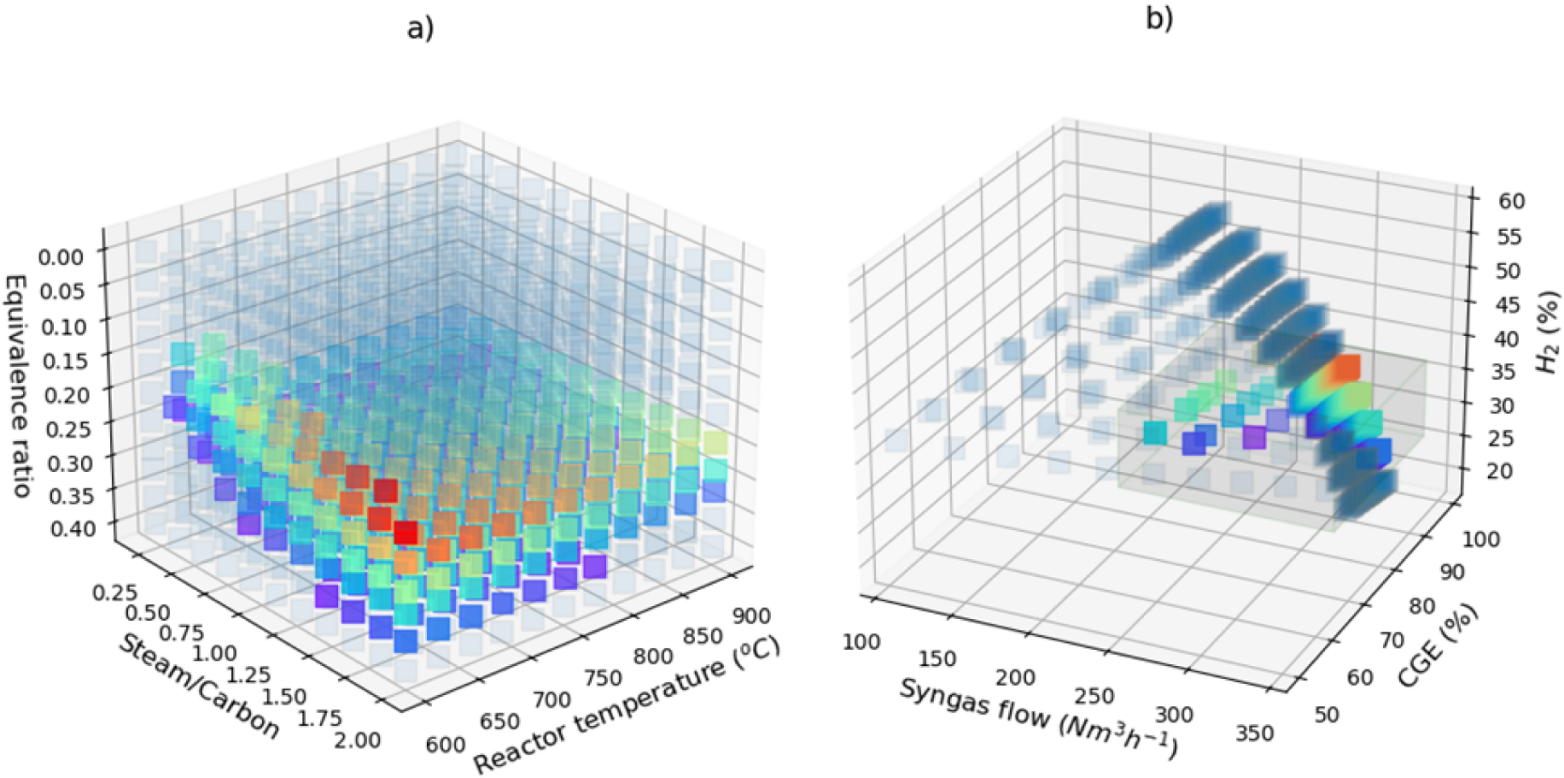
Operability analysis with energy demand constraint. a) Available Input Set (AIS), with feasible and desirable inputs (DIS) highlighted in color; b) Achievable Output Set (AOS), with the Desired Output Set (DOS) represented as a transparent gray polytope and fDOS points shown in corresponding colors.

Figure 9 presents the energy demand profile of the açaí seed gasification process as a function of the operational conditions (*T*, ER, S/B ratio), within the optimized region, allowing a direct comparison between the two scenarios (with/without constraint). The main implication of imposing the energy demand constraint lies in the industrial feasibility of the process. In the first case, where energy demand was not limited, a higher hydrogen concentration was obtained, which may be advantageous for specific applications. However, this approach resulted in a significant increase in energy demand, reaching a maximum of 444 MJ h^–1^, which may compromise economic viability depending on energy costs and availability. In contrast, in the second case, by considering only points with Heat Duty <0, the optimization favored more sustainable conditions, reducing the need for external energy consumption and making the process more attractive for industrial implementation.

**Figure 9:**
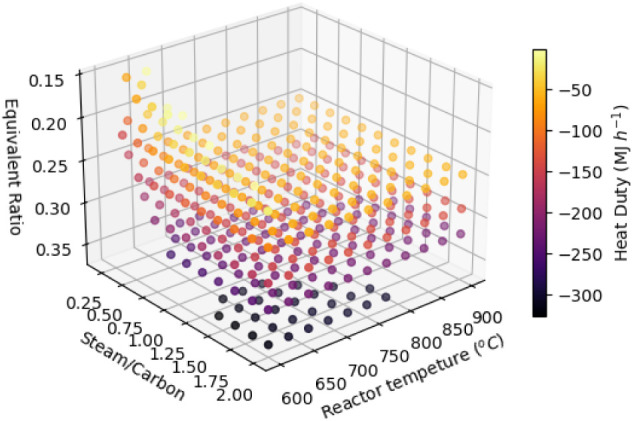
Energy demand profile of the açaí seed gasification process as a function of combustion temperature, equivalence ratio, and steam-to-biomass ratio. Operability analysis is performed with energy constraint, with heat duty mapped over the DIS region.

To meet the energy demand constraint, the main variable affected was the ER, which played a fundamental role in improving the system’s energy efficiency. However, this adjustment directly impacted the composition of the synthesis gas. The increased amount of air fed into the system resulted in a reduction in hydrogen concentration, making the syngas less enriched in this component. In addition, although cold gas efficiency (CGE) was relatively preserved, there was a slight negative impact, evidenced by a small reduction in its maximum values. This compromise in CGE reflects the necessary balance between energy efficiency and final product quality, a crucial factor for the industrial implementation of the gasification process.

Figure 10 presents the operability analysis of the gasification process under energy constraints. As in the previous case, the green surface represents the polytope obtained from the simulated process results, while the red region defines the desired output set. The overlap between the feasible polytope and the desired set is visibly greater than the scenario without energy constraint, reflecting higher operability under the imposed conditions.

**Figure 10:**
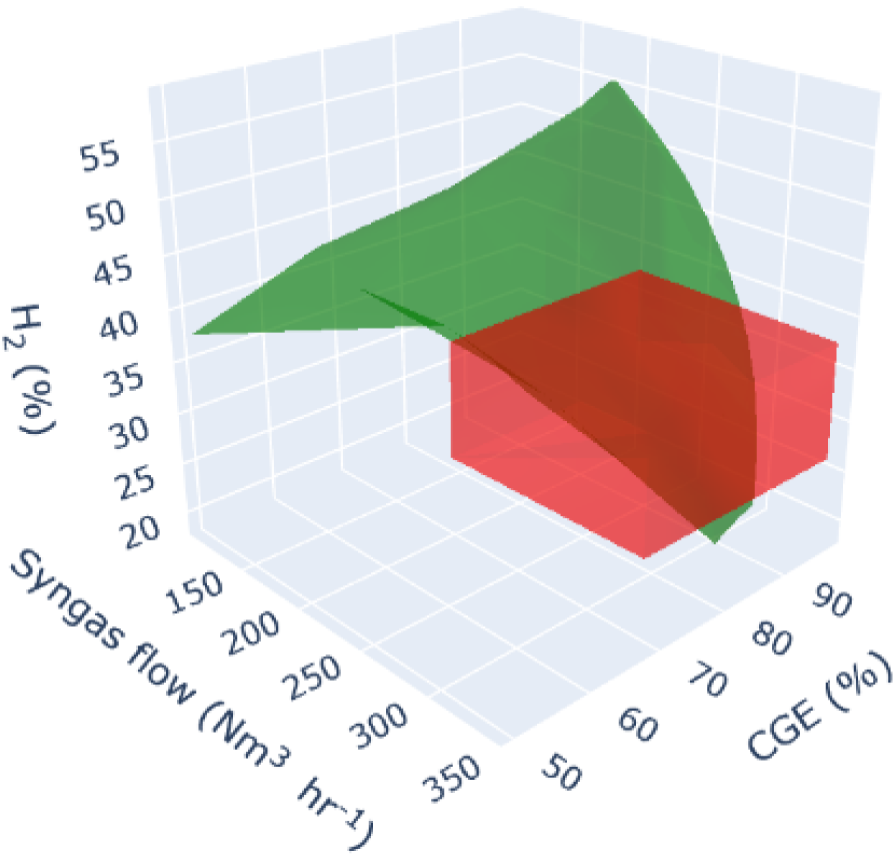
Operability map of the gasification process with energy constraint (Heat Duty < 0): intersection between AOS (green polytope) and DOS (red polytope). Operability index = 30.2%.

The requirement of autothermal operation (heat duty <0) reduces the volume of the polytope, mainly in the direction of hydrogen composition, by excluding thermally unfeasible regions. However, this constraint concentrates the operation in an output space zone where conversion efficiency and gas flow are favored. From a quantitative perspective, this greater compatibility is reflected in an operability index of 30.2%. This demonstrates that nearly one-third of the defined targets can be achieved without the need for external energy input, highlighting the process’s potential to operate energetically self-sufficiently under optimized conditions.

The analyses allowed the identification of the main implications of the energy demand constraint on the optimization of the gasification process. The most significant finding is the direct influence this constraint exerts on the system’s operability and the synthesis gas composition. In the first case, where energy demand was not limited, a higher hydrogen concentration in the syngas was achieved, which may be advantageous for specific industrial applications. However, this approach resulted in a significantly high energy demand, reaching 443.5 MJ h^–1^, which may compromise the economic viability of the process in scenarios where energy cost is a limiting factor. In contrast, in the second case, by considering only points with Heat Duty <0, the optimization favored a more self-sufficient process, reducing the need for external energy supply and increasing the operability index from 13% to 30.2%, indicating a more efficient system under the energy criterion.

## 4 Conclusions

This study addressed the gasification of açaí pit biomass through a thermodynamic modeling approach. A process model was developed and implemented in Aspen Plus to simulate the steady-state behavior of the system, and an operability analysis was conducted to identify feasible and optimal operating regions based on multiple performance criteria. The modeling approach considered reactor temperature, steam-to-biomass (S/B) ratio, and equivalence ratio (ER) as key control variables. The results revealed that both the equivalence ratio and the steam-to-biomass ratio significantly influence syngas composition, volumetric flow rate, and cold gas efficiency (CGE). Higher ER values favored complete oxidation reactions, increasing CO_2_ and N_2_ concentrations but reducing the hydrogen content. In contrast, increasing S/B ratios promoted hydrogen production via the water–gas shift and reforming reactions while impacting CO levels through competing reaction mechanisms. Operability analysis provided valuable insights into the optimal regions of operation, considering multiple objectives simultaneously. Higher hydrogen concentrations (up to 58.6 mol %) were achieved without energy constraints but at the expense of elevated energy demand, reaching up to 444 MJ h^–1^. When energy demand was restricted (Heat Duty <0), the operability index increased from 13% to 30.2%, demonstrating that a considerable portion of the desired targets can be achieved under energetically self-sustaining conditions. In this constrained scenario, CGE remained high (up to 98%), although the hydrogen content in the syngas decreased. These findings highlight the trade-offs between energy efficiency and product quality in biomass gasification and reinforce the importance of operability-based optimization. The methodology adopted in this work proved effective in identifying feasible and optimal operating regions, contributing to the advancement of gasification process design with a focus on energetic autonomy and industrial applicability.

## Supplementary Material



## Notes

Additional tables, including Table S1—Main chemical reactions in biomass gasification, and Table S2—Comparison of syngas lower heating value (LHV) calculated by the proposed equation and obtained from Aspen Plus V11 as a function of the equivalence ratio (ER) (PDF)
