# Supplementary material for: Modeling and Simulation of the Gasification of Euterpe oleracea Waste: Optimization of Hydrogen Production and Energy Potential through Operability Analysis
Source: ACS Omega. 2026 Jun 1;11(23):34034–46. doi: 10.1021/acsomega.6c01087 (PMC13280886; doi:10.1021/acsomega.6c01087)
Supplement: Supplementary file 1 [file ao6c01087_si_001.pdf]

Supporting information

Modeling and simulation of the gasification of *Euterpe oleracea* waste: optimization of hydrogen production and energy potential through operability analysis

Cassiano M. Musial[a,b], Júnior Staudt[c], Augusto Verdi Reichert[a], Edson A. da Silva[a], Caroline Ribeiro [a], Fernando V. Lima[b] and Carlos E. Borba[a]

[a] Postgraduate Program in Chemical Engineering, West Parana State University, Campus Toledo, Faculdade St. 645, Jd. La Salle, 85903-000, Toledo, PR, Brazil.

[b] Department of Chemical and Biomedical Engineering, West Virginia University, Engineering Sciences Building, 1306 Evansdale Dr, Morgantown, WV 26506, United States.

[c] Laboratory of Chemical Process Engineering, Technical University of Munich, Campus Straubing for Biotechnology and Sustainability, 94315 Straubing, Germany

Corresponding Author E-mail: cassiano.musial@unioeste.br

Table S1. Main chemical reactions in biomass gasification

**Name**	**Reaction**	**ΔH298 K (kJ/mol)**
**Partial oxidation of the carbon**	$C+\frac{1}{2}O_2 \rightarrow CO$	-112
**Complete oxidation of the carbon**	$C+O_2 \rightarrow CO_2$	-393
**Boudouard reaction**	$C+CO_2 \rightleftarrows 2CO$	+172
**Water-gas shift reaction**	$\mathrm{CO}+H_2O \rightleftarrows CO_2+H_2$	-41
**Steam gasification**	$C+H_2O \rightleftarrows \mathrm{CO}+H_2$	+131
**Oxidation of the hydrogen**	$H_2+\frac{1}{2}O_2 \rightarrow H_2O$	-242
**Hydrogasification reaction**	$C+2H_2 \rightleftarrows CH_4$	-74
**Steam reforming methane**	$CH_4+H_2O \rightleftarrows \mathrm{CO}+3H_2$	+206
**Dry reforming methane**	$CH_4+CO_2 \rightleftarrows 2\mathrm{CO}+2H_2$	+247

Table S2. Comparison of syngas lower heating value (LHV) calculated by the proposed equation and obtained from Aspen Plus V11 as a function of the equivalence ratio (ER).

S/B	LHV (calculated)	LHV (Aspen v11)	RMSE
0,00	9,120	9,122	0,05
0,04	8,341	8,343	0,05
0,04	8,339	8,341	0,05
0,08	7,638	7,641	0,05
0,12	6,999	7,002	0,05
0,16	6,416	6,419	0,06
0,20	5,879	5,883	0,06
0,24	5,384	5,387	0,06
0,28	4,926	4,929	0,06
0,31	4,499	4,502	0,05
0,35	4,102	4,104	0,05
0,39	3,730	3,732	0,05
